# Supplementary material for: Genetics of osteopontin in patients with chronic kidney disease: The German Chronic Kidney Disease study
Source: PLoS Genet. 2022 Apr 6;18(4):e1010139. doi: 10.1371/journal.pgen.1010139 (PMC9015153; doi:10.1371/journal.pgen.1010139)
Supplement: S1 Fig — (PDF) [file pgen.1010139.s001.pdf]

S1 Figure: Flow chart showing exclusion of patients and analysis sets.

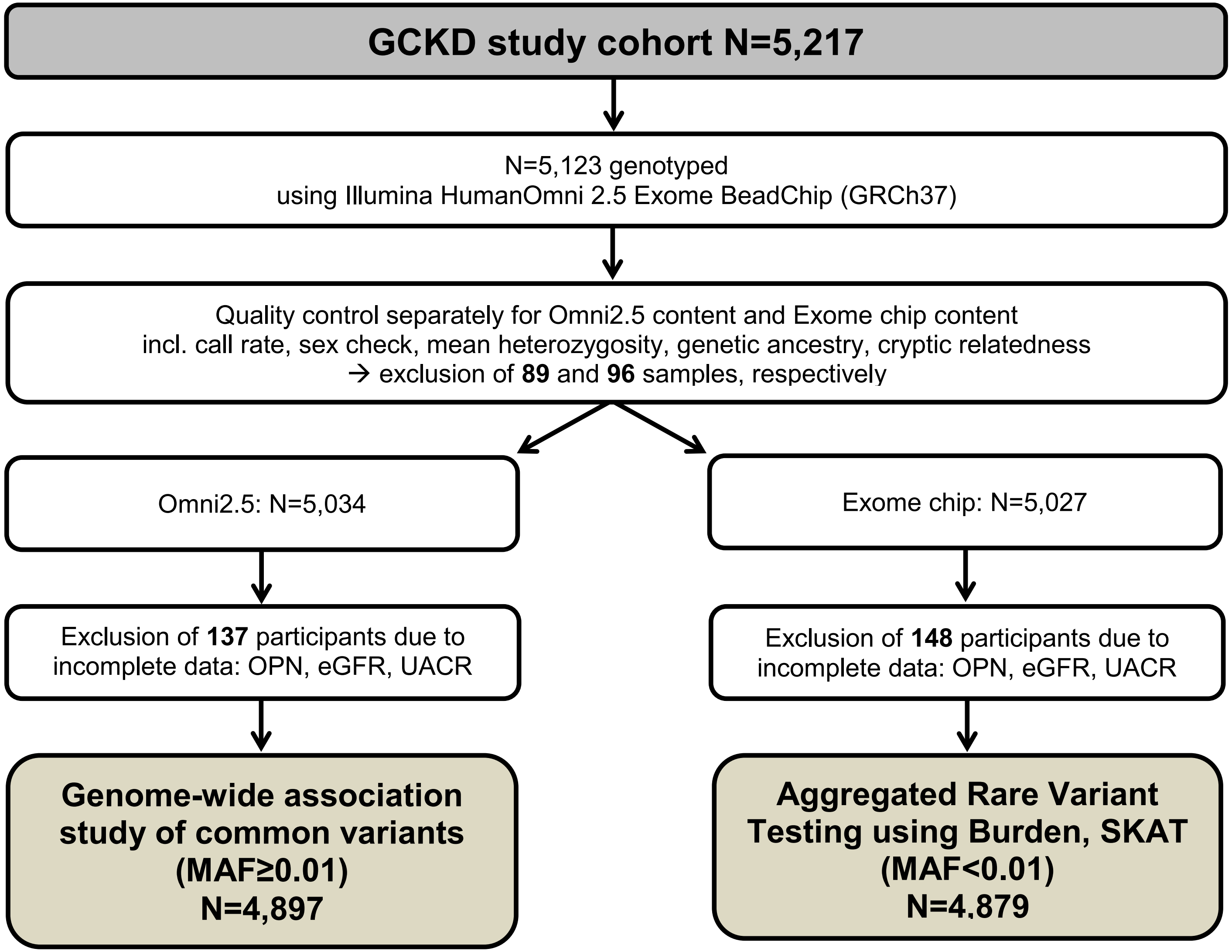

OPN: osteopontin; eGFR: estimated glomerular filtration rate; UACR: urinary albumin to creatinine ratio; SKAT: sequence kernel association test; MAF: minor allele frequency
