# Supplementary material for: Genetics of osteopontin in patients with chronic kidney disease: The German Chronic Kidney Disease study
Source: PLoS Genet. 2022 Apr 6;18(4):e1010139. doi: 10.1371/journal.pgen.1010139 (PMC9015153; doi:10.1371/journal.pgen.1010139)

S2 Figure: Osteopontin (OPN, ng/mL) measurements in GCKD.

Osteopontin	N	mean	min	max	p25	p50	p75
total	5154	34.76	4.72	247.09	20.67	29.25	41.85

Legend:
eGFR (mL/min/1.73m^2) categories: G1/2≥60, G3a=45-59 G3b=30-44 G4/5<30
UACR (mg/g) categories: A1<30, A2=30-300, A3>300
red line: overall median OPN level (29.2 ng/mL)

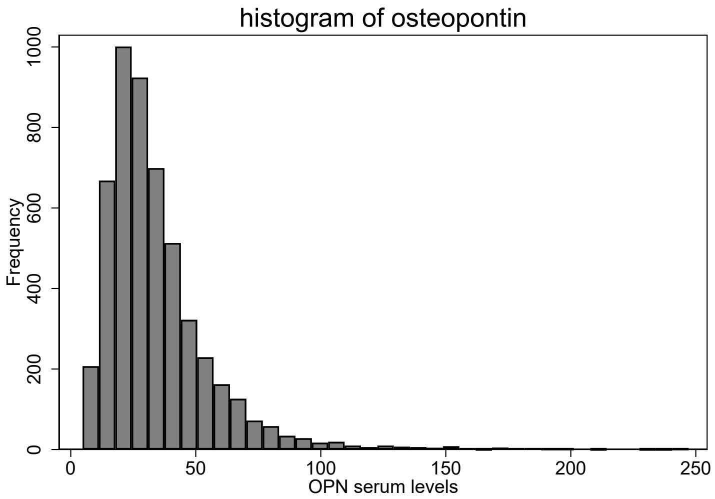

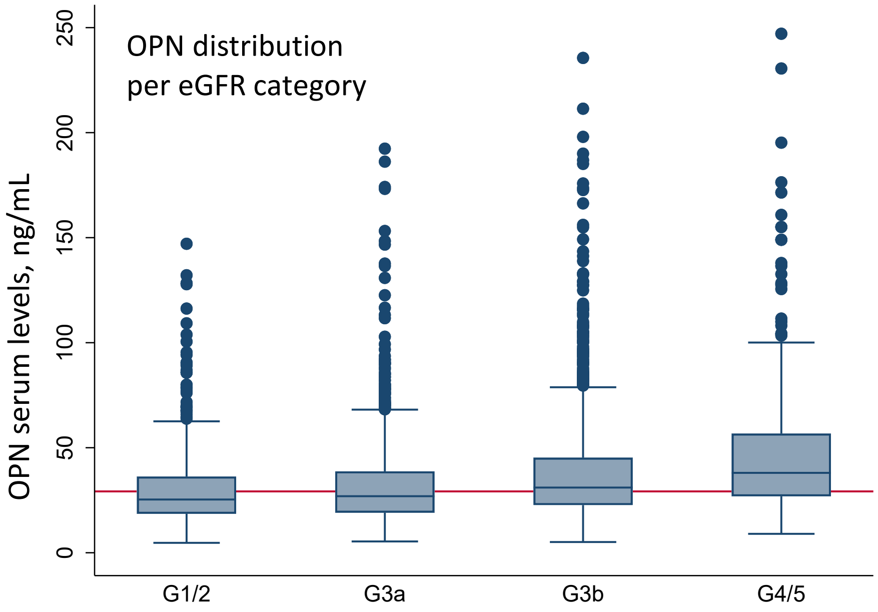

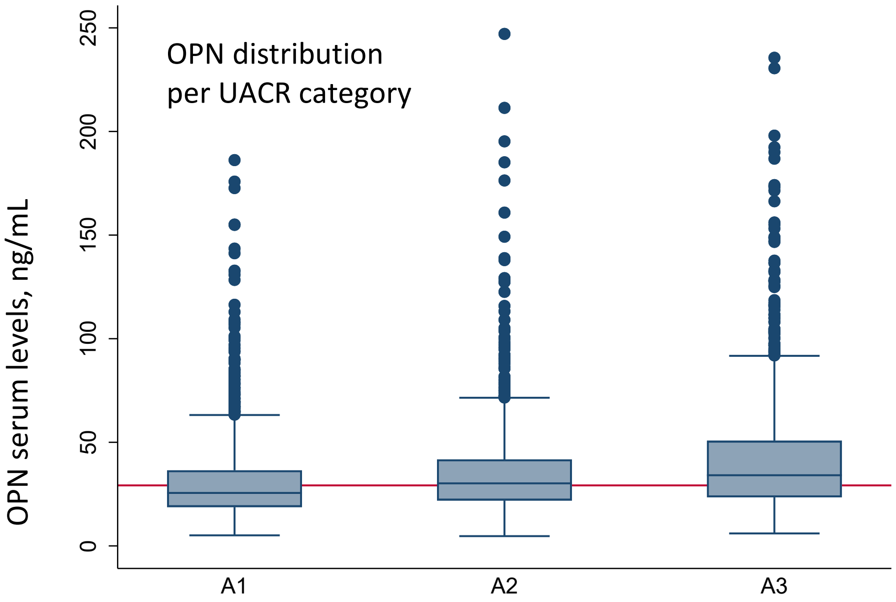

Supplement: S2 Fig — (PDF) [file pgen.1010139.s002.pdf]
