# Supplementary material for: Genetics of osteopontin in patients with chronic kidney disease: The German Chronic Kidney Disease study
Source: PLoS Genet. 2022 Apr 6;18(4):e1010139. doi: 10.1371/journal.pgen.1010139 (PMC9015153; doi:10.1371/journal.pgen.1010139)
Supplement: S3 Fig — (PDF) [file pgen.1010139.s003.pdf]

S3 Figure: Quantile-Quantile plot of results from GWAS of log_2(OPN).

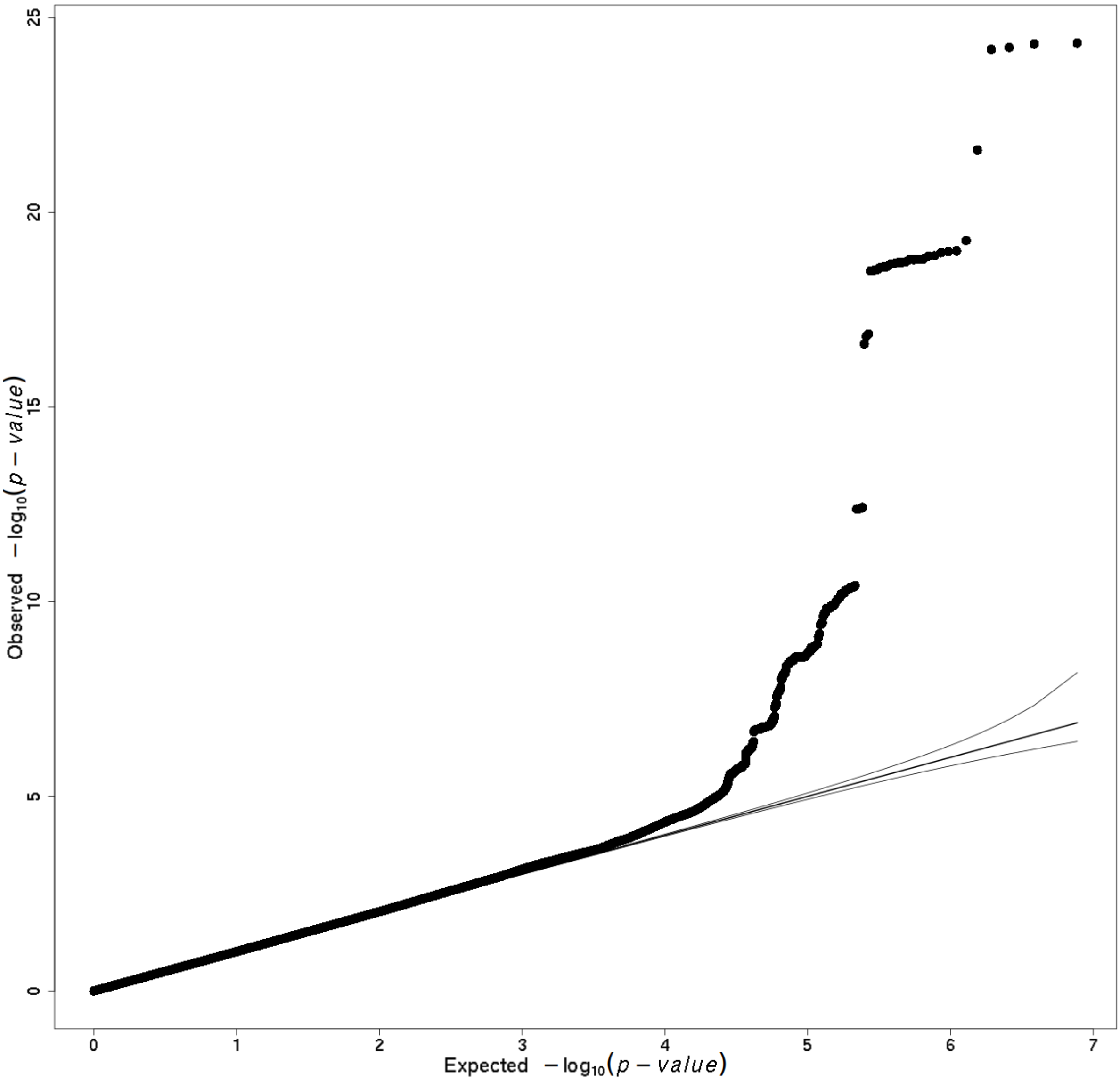

Association analysis based on imputed dosage data (additive genetic model) was adjusted for age, sex, log(eGFR), and log(UACR).

Results filtered for MAF≥0.01 are presented: inflation factor λ=1.01.
