# Supplementary material for: Genetics of osteopontin in patients with chronic kidney disease: The German Chronic Kidney Disease study
Source: PLoS Genet. 2022 Apr 6;18(4):e1010139. doi: 10.1371/journal.pgen.1010139 (PMC9015153; doi:10.1371/journal.pgen.1010139)
Supplement: S4 Fig — (PDF) [file pgen.1010139.s004.pdf]

S4 Figure: Manhattan plot of results from GWAS of log_2(OPN).

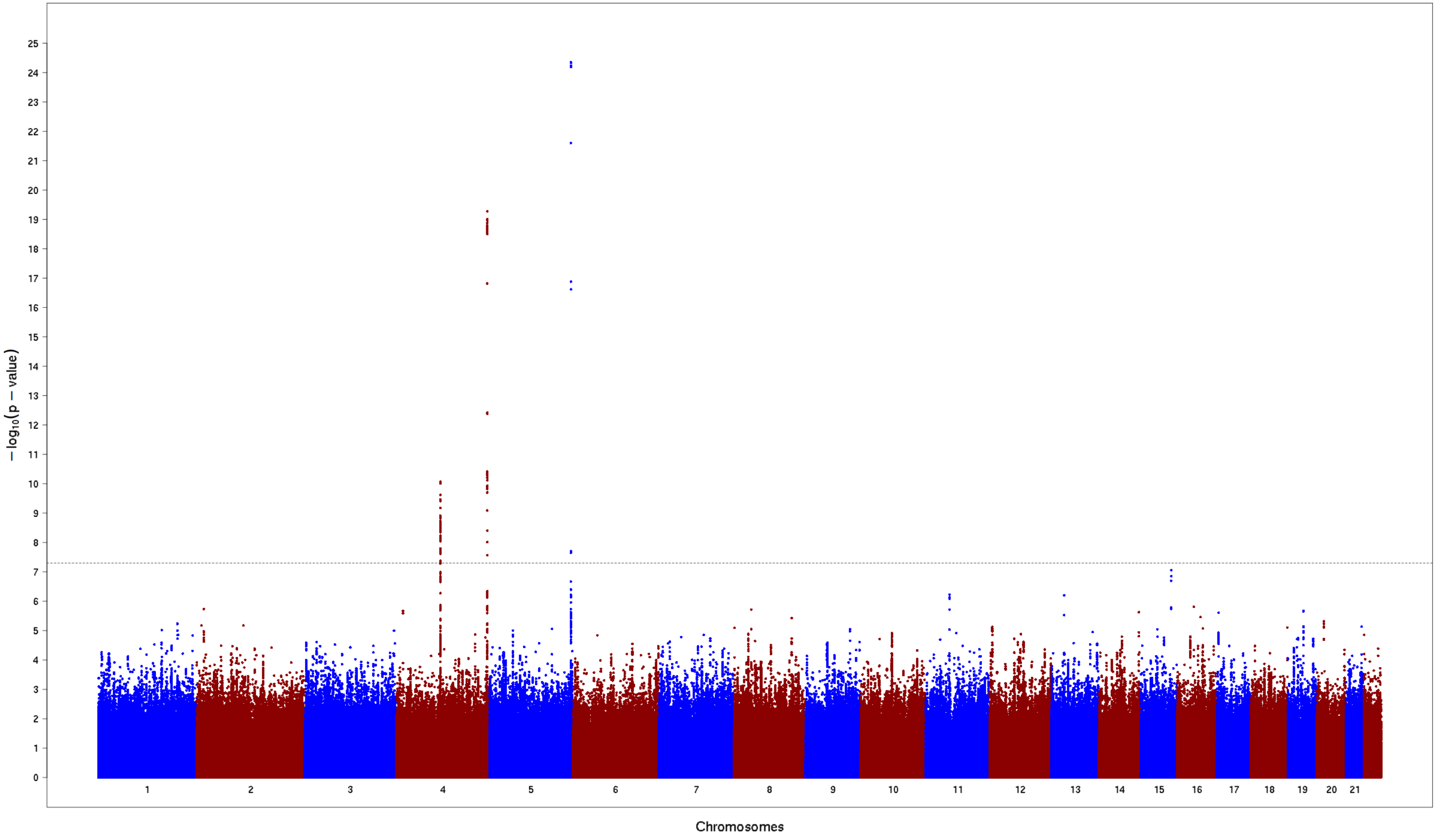

x-axis: chromosomal position (GRCh37); y-axis: $-log_{10}$-transformed p-values; grey line: genome-wide significance level (5.0E-08). Association analysis based on imputed dosage data (additive genetic model) was adjusted for age, sex, log(eGFR), and log(UACR). Results were filtered for MAF≥0.01.
