# Supplementary material for: Genetics of osteopontin in patients with chronic kidney disease: The German Chronic Kidney Disease study
Source: PLoS Genet. 2022 Apr 6;18(4):e1010139. doi: 10.1371/journal.pgen.1010139 (PMC9015153; doi:10.1371/journal.pgen.1010139)
Supplement: S5 Fig — (PDF) [file pgen.1010139.s005.pdf]

S5 Figure: Regional association plots obtained in the course of conditional analysis to identify independent signals.

Chromosome 4 | **Chromosome 5**

STEP 0: unconditional analysis

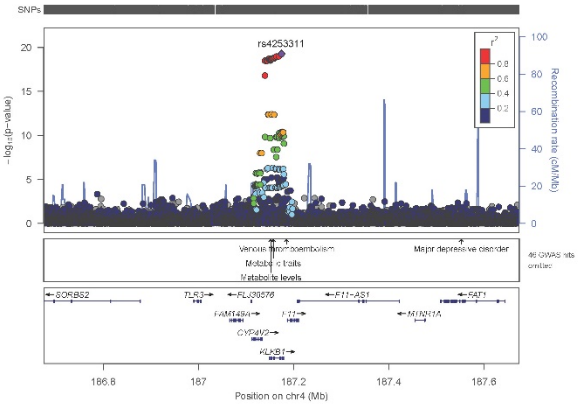

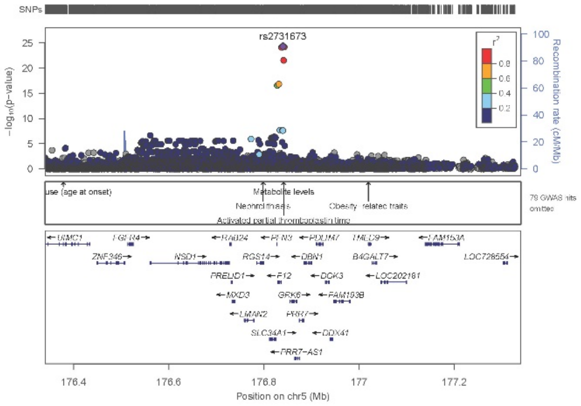

STEP 1: conditional analysis conditioning on SNPs with lowest p-value identified in step 0

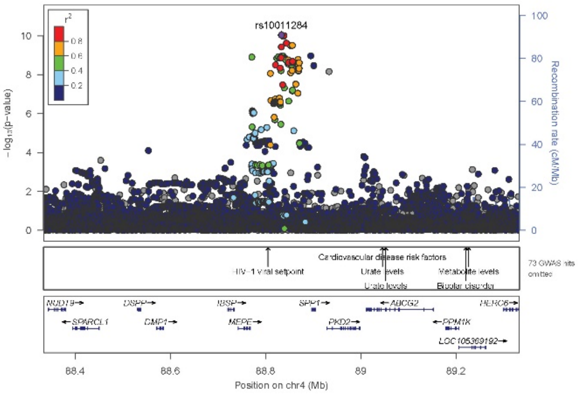

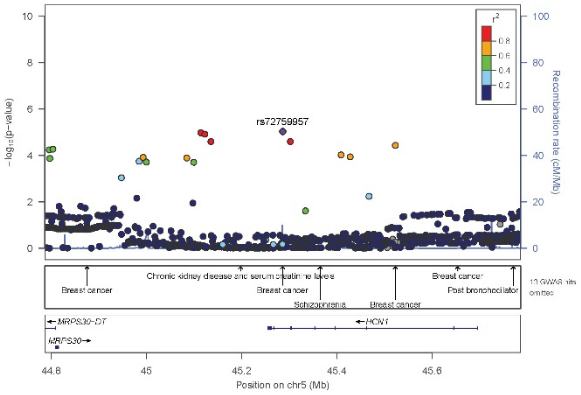

STEP 2: conditional analysis conditioning on SNPs with lowest p-value identified in steps 0 and 1

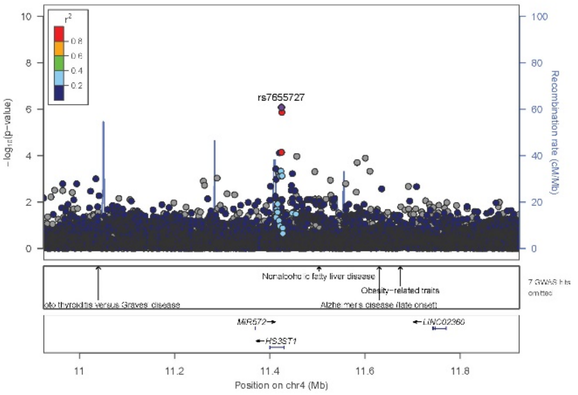

Association p-values for the SNP with lowest p-value across the chromosome:

Step	Condition	top SNP	Position (Build b37)	p-value
0	- (uncond.)	rs4253311	4:187174683	**5.29E-20**
1	rs4253311	rs10011284	4:88833389	**9.12E-11**
2	rs4253311, rs10011284	rs7655727	4:11424957	8.36E-07
0	- (uncond.)	rs2731673	5:176839898	**4.5E-25**
1	rs2731673	rs72759957	5:45286288	9.41E-06

For each chromosome with genome-wise significant associations, a conditional analysis run was the SNP with the lowest association p-value across the chromosome. This procedure was repeated until no further genome-wide significant association was observed. Step 0 corresponds to the unconditional analysis.
