# Supplementary material for: Genetics of osteopontin in patients with chronic kidney disease: The German Chronic Kidney Disease study
Source: PLoS Genet. 2022 Apr 6;18(4):e1010139. doi: 10.1371/journal.pgen.1010139 (PMC9015153; doi:10.1371/journal.pgen.1010139)
Supplement: S6 Fig — (PDF) [file pgen.1010139.s006.pdf]

S6 Figure: Levels of osteopontin (log_2-transformed) in the overall cohort and across genotypes of discovered SNPs in GCKD.

(A) GCKD overall

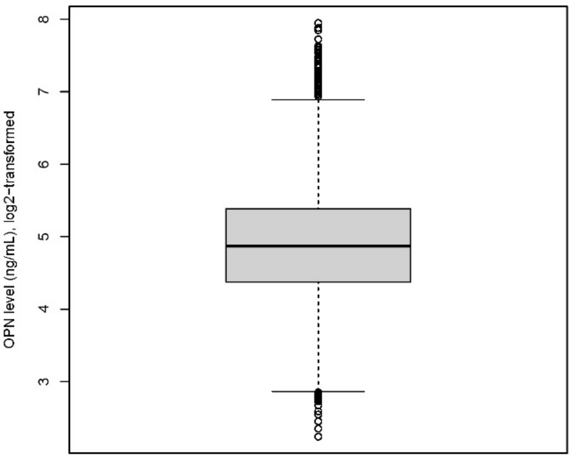

(B) rs10011284 (4:88833389)

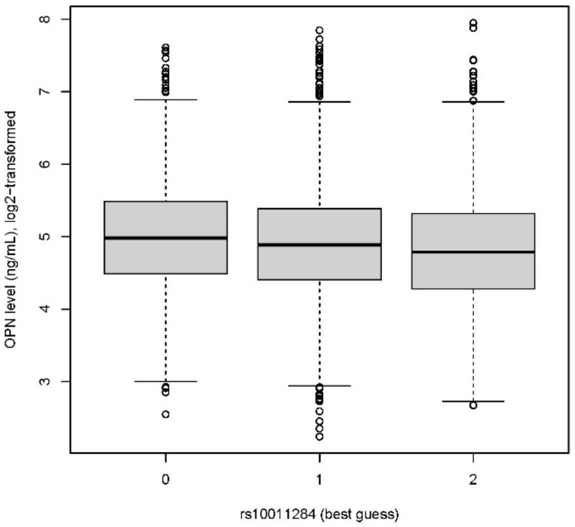

(C) rs4253311 (4:187174683)

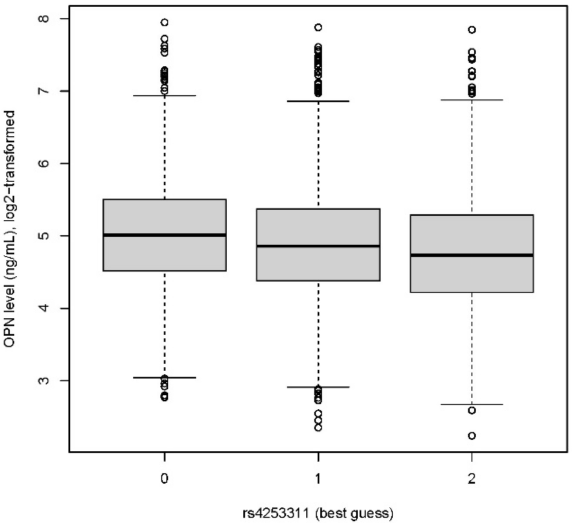

(D) rs2731673 (5:176839898)

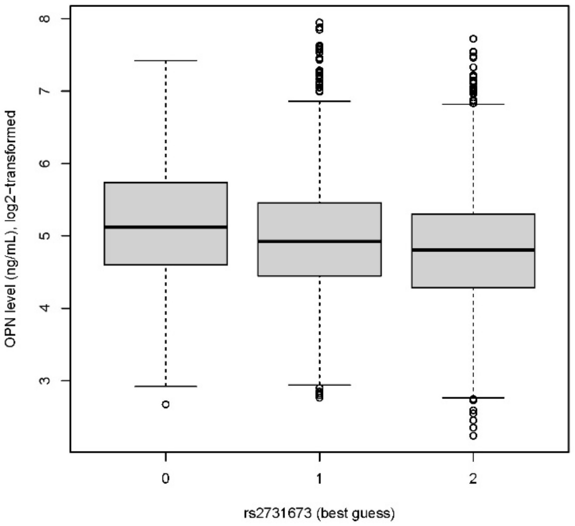

For the purpose of presentation, dosages of SNPs were transformed into best guess genotypes.
