# Supplementary material for: Genetics of osteopontin in patients with chronic kidney disease: The German Chronic Kidney Disease study
Source: PLoS Genet. 2022 Apr 6;18(4):e1010139. doi: 10.1371/journal.pgen.1010139 (PMC9015153; doi:10.1371/journal.pgen.1010139)
Supplement: S7 Fig — (PDF) [file pgen.1010139.s007.pdf]

S7 Figure: Regional association plots for the region around rs2731673 on chromosome 5.

(A) GCKD study (discovery)

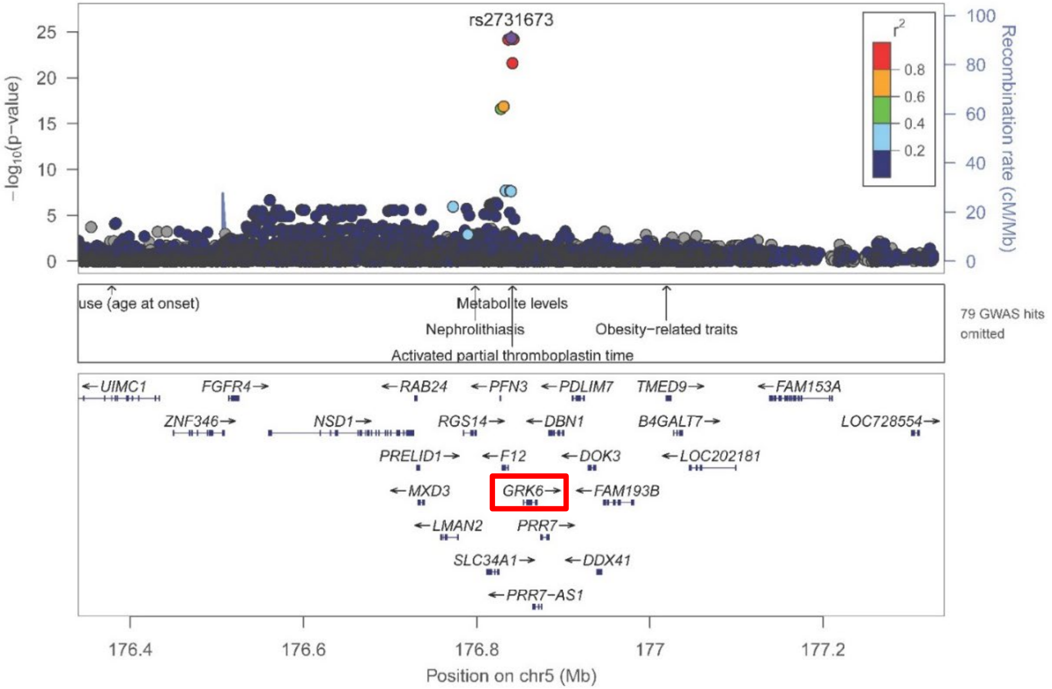

(B) YFS cohort (replication)

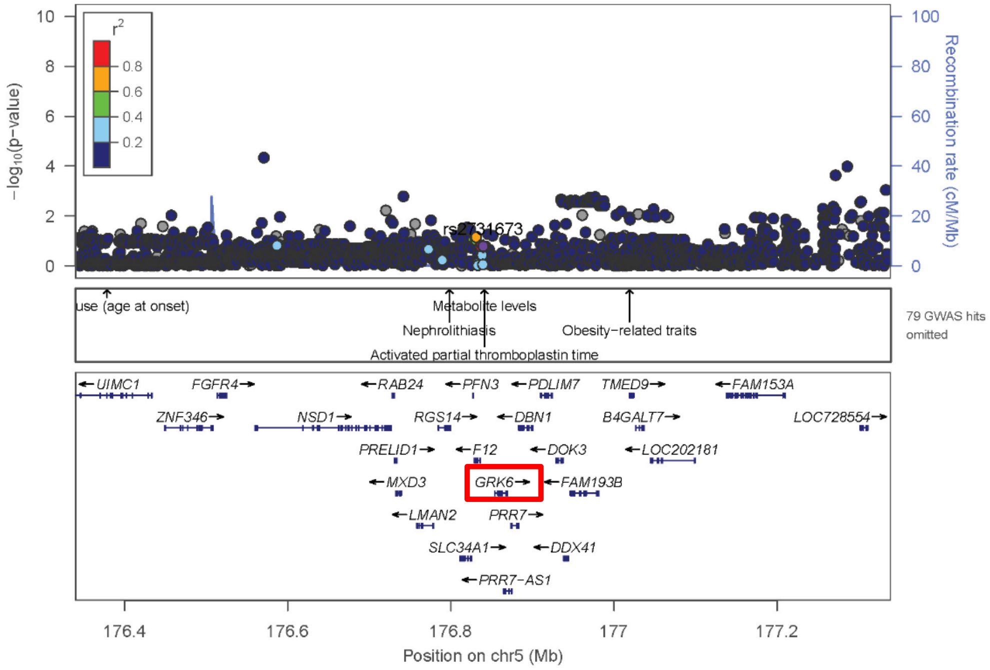

x-axis: chromosomal position; y-axis: left - association p-values (-log$_{10}$-transformed), right – genetic recombination rates

Plot shows the association results around the most strongly associated SNP of this region (purple diamond); SNP colors reflect LD correlation (r^2) using 1000G EUR population as reference.
