# Supplementary material for: Genetics of osteopontin in patients with chronic kidney disease: The German Chronic Kidney Disease study
Source: PLoS Genet. 2022 Apr 6;18(4):e1010139. doi: 10.1371/journal.pgen.1010139 (PMC9015153; doi:10.1371/journal.pgen.1010139)
Supplement: S8 Fig — (PDF) [file pgen.1010139.s008.pdf]

S8 Figure: Effects of rs10011284 and rs4253311 (chromosome 4) on OPN levels.

(A) rs10011284 (4:88833389)

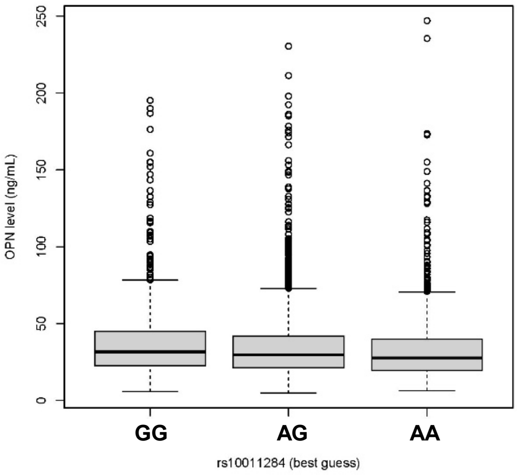

(B) rs4253311 (4:187174683)

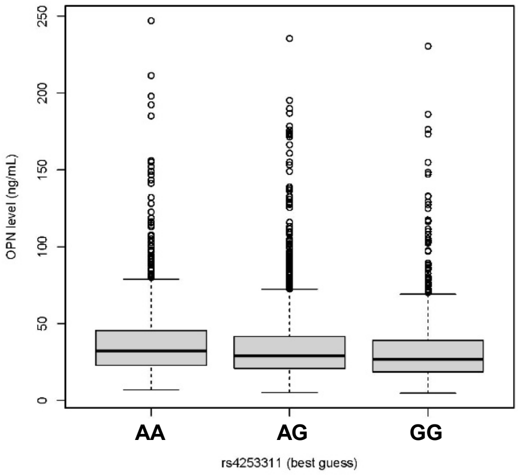

(C) any combination of both SNPs

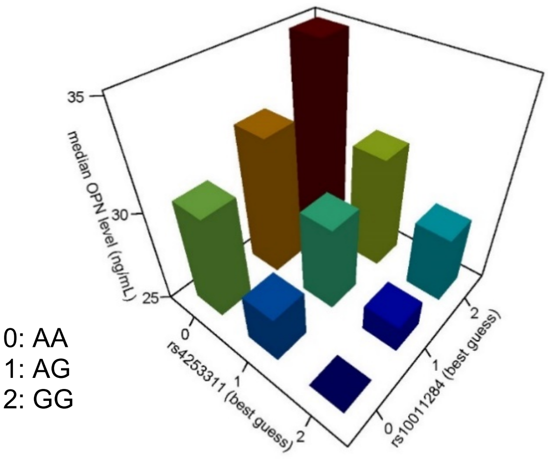

0: AA
1: AG
2: GG

		rs4253311 (best guess)			Total
		**GG**	**AG**	**AA**	
**rs10011284 (best guess)**	**AA**	236	475	228	939
	**AG**	585	1193	588	2366
	**GG**	398	784	410	1592
**Total**		1219	2452	1226	4897

For the presentations, dosage information was transformed into best guess genotypes. The accompanying table presents observed frequencies of alleles and their combinations in the GCKD study. A formal test for multiplicative interaction of both SNPs on OPN levels was not significant (p=9.96E-01).

Human genome build: GRCh37
