# Supplementary material for: Genetics of osteopontin in patients with chronic kidney disease: The German Chronic Kidney Disease study
Source: PLoS Genet. 2022 Apr 6;18(4):e1010139. doi: 10.1371/journal.pgen.1010139 (PMC9015153; doi:10.1371/journal.pgen.1010139)
Supplement: S9 Fig — (PDF) [file pgen.1010139.s009.pdf]

S9 Figure: Relationship of selected GO terms.

(A) Reference: GO cellular component

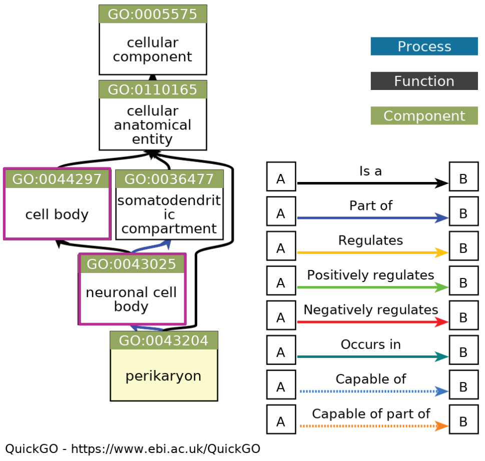

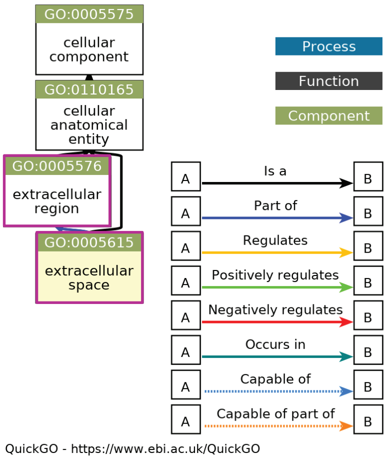

enriched category:

data base query: 21-05-31

(B) Reference: GO molecular function

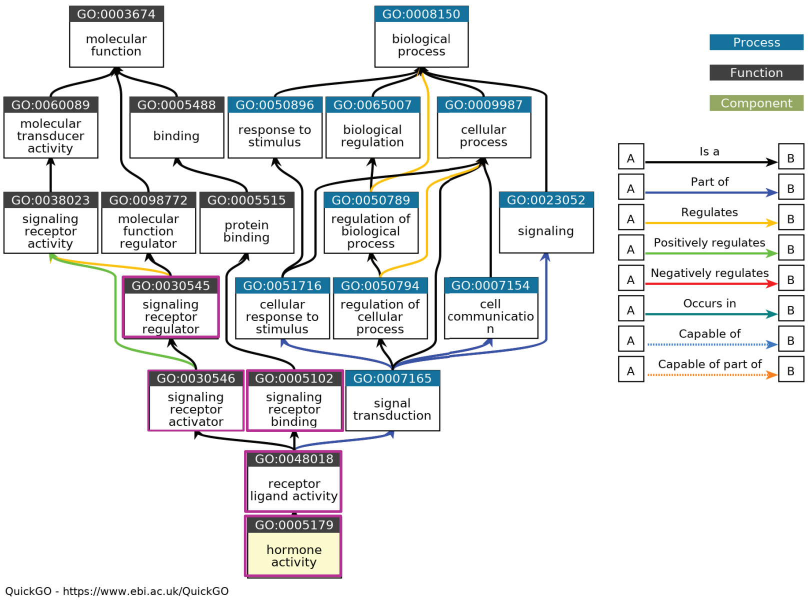

enriched category:

data base query: 21-05-31
