# Supplementary material for: Genetics of osteopontin in patients with chronic kidney disease: The German Chronic Kidney Disease study
Source: PLoS Genet. 2022 Apr 6;18(4):e1010139. doi: 10.1371/journal.pgen.1010139 (PMC9015153; doi:10.1371/journal.pgen.1010139)
Supplement: S10 Fig — (PDF) [file pgen.1010139.s010.pdf]

S10 Figure: Comparing summary statistics for the *KLKB1* locus from OPN GWAS (unconditional statistics, A) and the UK Biobank phenotype deep venous thrombosis (DVT, conditional statistics, B).

(A) OPN GWAS results for *KLKB1* locus in GCKD study

(B) DVT GWAS results for *KLKB1* locus in UK Biobank after conditioning on rs4253399

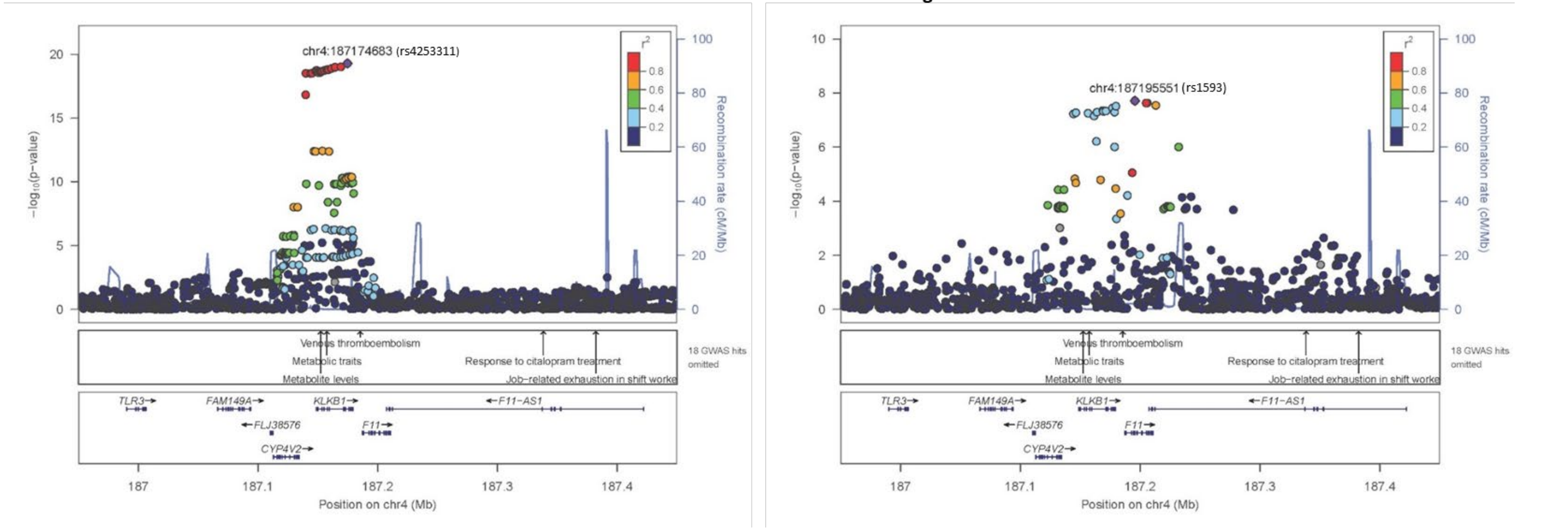

In conditional analysis, two independent SNPs for the *KLKB1* region were identified for the trait DVT in UK Biobank: 1) rs4253399 and 2) rs1593. Using summary statistics adjusted for rs4253399 of DVT (UK Biobank, B), colocalization with OPN GWAS signal (A) was observed (H4: p12=0.96).
