# Supplementary material for: Genetics of osteopontin in patients with chronic kidney disease: The German Chronic Kidney Disease study
Source: PLoS Genet. 2022 Apr 6;18(4):e1010139. doi: 10.1371/journal.pgen.1010139 (PMC9015153; doi:10.1371/journal.pgen.1010139)
Supplement: S1 Methods — (DOCX) [file pgen.1010139.s011.docx]

**S1 Methods**: The Cardiovascular Risk in Young Finns Study (YFS) cohort.

***Study population***

The Cardiovascular Risk in Young Finns Study (YFS) is an ongoing, prospective, multi-center study of atherosclerosis precursors in Finnish children and adolescents initiated in 1980. All study details have been described before [1]. Briefly, the first cross-sectional survey was conducted in 1980, when 3,596 participants, were randomly chosen from the national population register of Finland. Regular follow-up visits are conducted. Written informed consent was obtained from all participants or if under the age of 18 years obtained from a parent and/ or legal guardian.

All documents and protocols were designed corresponding to the proposal by the World Health Organization and were approved by the 1st Ethical Committee of the Hospital District of Southwest Finland and by local ethical committees (Regional Ethics Committee of the Expert Responsibility area of Tampere University Hospital, Helsinki University Hospital Ethical Committee of Medicine, The Research Ethics Committee of the Northern Savo Hospital District and Ethics Committee of the Northern Ostrobothnia Hospital District). The study was conducted in accordance with the Helsinki declaration.

***OPN measurement***

Plasma OPN was first measured in 2007 by enzyme-linked immunosorbent assay (Human Osteopontin Quantikine kit, R&D Systems, USA) from samples thawed for the first time for the assay. The inter-assay coefficient of variation (CV) of the Human Osteopontin Quantikine kit was 7.2% at a nominal concentration of 60 g/mL during this study [2].

The distribution and determinants of OPN were studied in a randomized cohort of 1,817 young adults (aged 30-45 years) without clinical symptoms of atherosclerosis. Here, the mean of the OPN concentrations was 60.7 μg/mL (SD: 15.6) in men and 51.7 μg/mL (SD: 16.0) in women [2].

***Genotyping, quality control and imputation***

Genomic DNA was extracted from peripheral blood leukocytes using a commercially available kit and Qiagen BioRobot M48 Workstation according to the manufacturer’s instructions (Qiagen, Hilden, Germany). Genotyping was done for 2,556 samples using a custom build Illumina Human 670k BeadChip at the Welcome Trust Sanger Institute. Genotypes were called using Illumina’s clustering algorithm. Fifty-six samples failed the Sanger genotyping pipeline due to QC criteria (i.e., duplicated samples, heterozygosity, low call rate, or Sequenom fingerprint discrepancy) and were excluded. From the remaining 2,500 samples, one sample failed gender check, three were removed due to low genotyping call rate (<0.95) and 54 samples for possible relatedness (pi-hat >0.2). 11,766 SNPs were excluded based on Hardy-Weinberg equilibrium test (p ≤1e-06), 7,746 SNPs failed missingness test (call rate <0.95) and 34,596 SNPs failed frequency test (MAF <0.01). After quality control there were 2,442 samples and 546,677 genotyped SNPs available for further analysis. Genotype imputation was performed using Minimac3 [3] and 1000G phase3 reference set on the Michigan Imputation Server. Autosomes and sex chromosomes were phased using Eagle [4] and SHAPEIT [5] respectively. Genomic positions base on human genome build GRCh37.

***Analysis set in the replication effort***

In this replication effort, data of 1,979 participants of the YFS cohort with genetic data and measurements on plasma OPN were used. The mean age was 38 years (SD: 5) and the mean eGFR was 92.62 mL/min/1.73m^2^ (SD: 20.74). The distribution of the OPN concentrations in this cohort is presented in the following table:

| Osteopontin  (µg/mL) | N | Mean | Min | Max | P25 | P50 | P75 |
| --- | --- | --- | --- | --- | --- | --- | --- |
| Total | 1,979 | 56.42 | 4 | 181 | 46 | 55 | 66 |

***References***

1. Akerblom HK, Viikari J, Uhari M, Räsänen L, Byckling T, Louhivuori K, et al. Atherosclerosis precursors in Finnish children and adolescents. I. General description of the cross-sectional study of 1980, and an account of the children's and families' state of health. Acta paediatrica Scandinavica Supplement. 1985;318:49-63. Epub 1985/01/01. doi: 10.1111/j.1651-2227.1985.tb10082.x. PubMed PMID: 3879091.

2. Wendelin-Saarenhovi M, Oikonen M, Loo BM, Juonala M, Kähönen M, Viikari JS, et al. Plasma osteopontin is not associated with vascular markers of subclinical atherosclerosis in a population of young adults without symptoms of cardiovascular disease. The Cardiovascular Risk in Young Finns Study. Scandinavian journal of clinical and laboratory investigation. 2011;71(8):683-9. Epub 2011/10/25. doi: 10.3109/00365513.2011.621027. PubMed PMID: 22017169.

3. Das S, Forer L, Schonherr S, Sidore C, Locke AE, Kwong A, et al. Next-generation genotype imputation service and methods. Nat Genet. 2016;48(10):1284-7. doi: 10.1038/ng.3656. PubMed PMID: 27571263; PubMed Central PMCID: PMCPMC5157836.

4. Loh PR, Palamara PF, Price AL. Fast and accurate long-range phasing in a UK Biobank cohort. Nat Genet. 2016;48(7):811-6. Epub 2016/06/09. doi: 10.1038/ng.3571. PubMed PMID: 27270109; PubMed Central PMCID: PMCPMC4925291.

5. Delaneau O, Zagury JF, Marchini J. Improved whole-chromosome phasing for disease and population genetic studies. Nat Methods. 2013;10(1):5-6. Epub 2012/12/28. doi: 10.1038/nmeth.2307

nmeth.2307 PubMed PMID: 23269371.
