# Supplementary material for: Genetics of osteopontin in patients with chronic kidney disease: The German Chronic Kidney Disease study
Source: PLoS Genet. 2022 Apr 6;18(4):e1010139. doi: 10.1371/journal.pgen.1010139 (PMC9015153; doi:10.1371/journal.pgen.1010139)
Supplement: S1 Information — (DOCX) [file pgen.1010139.s012.docx]

**S1 Information**: List of institutions and investigators participating in the GCKD study.

**Current GCKD Investigators and Collaborators with the GCKD Study are:**

University of Erlangen: Kai-Uwe Eckardt, Heike Meiselbach, Markus P. Schneider, Mario Schiffer, Hans-Ulrich Prokosch, Barbara Bärthlein, Andreas Beck, André Reis, Arif B. Ekici, Susanne Becker, Dinah Becker-Grosspitsch, Ulrike Alberth-Schmidt, Birgit Hausknecht, Anke Weigel;

University of Freiburg: Gerd Walz, Anna Köttgen, Ulla T. Schultheiß, Fruzsina Kotsis, Simone Meder, Erna Mitsch, Ursula Reinhard;

RWTH Aachen University: Jürgen Floege, Turgay Saritas;

Charité, University Medicine Berlin: Elke Schaeffner, Seema Baid-Agrawal, Kerstin Theisen;

Hannover Medical School: Hermann Haller, Jan Menne;

University of Heidelberg: Martin Zeier, Claudia Sommerer, Johanna Theilinger

University of Jena: Gunter Wolf, Martin Busch, Rainer Paul;

Ludwig-Maximilians University of München: Thomas Sitter;

University of Würzburg: Christoph Wanner, Vera Krane, Antje Börner-Klein, Britta Bauer;

Medical University of Innsbruck, Division of Genetic Epidemiology: Florian Kronenberg, Julia Raschenberger, Barbara Kollerits, Lukas Forer, Sebastian Schönherr, Hansi Weissensteiner;

University of Regensburg, Institute of Functional Genomics: Peter Oefner, Wolfram Gronwald;

Institute of Medical Biometry, Informatics and Epidemiology, Medical Faculty, University of Bonn: Matthias Schmid, Jennifer Nadal.
