# Supplementary material for: Genetics of osteopontin in patients with chronic kidney disease: The German Chronic Kidney Disease study
Source: PLoS Genet. 2022 Apr 6;18(4):e1010139. doi: 10.1371/journal.pgen.1010139 (PMC9015153; doi:10.1371/journal.pgen.1010139)
Supplement: Attachment — Submitted filename: EditorReply_ReviewerComments.pdf [file pgen.1010139.s021.pdf]

Gregory S. Barsh, MD, PhD
Gregory P. Copenhaver, PhD
Editors-in-Chief

March 4th, 2022

Dear Prof. Barsh and dear Prof. Copenhaver,
Dear Reviewers,

We thank you all for your time and good assessment of our manuscript and are very happy to submit a revised version of our manuscript entitled "Genetics of osteopontin in patients with chronic kidney disease: the German Chronic Kidney Disease study" (PGENETICS-D-21-01377R1).

The revision addresses the final question of Reviewer 3 leading to a rephrasing of the respective sentence to clarify our statement on carried out conditional analyses. For details, please see our response below.

In addition, we used the opportunity to insert the link to the data availability statement (page 29 of the manuscript) from which complete summary statistics from the GCKD study can be obtained. Also we have identified a small error in the conditional colocolization analysis, and updated the values in Supporting Table 7, as well as a p-value in the manuscript file (page 9, line 220) and supporting material file. However, non of our conclusions or any of our message have changed due to this small update.

Other than the supporting tables and the supporting material file, we are submitting two versions of our altered manuscript: 1) a corrected manuscript version with changes marked in track changes (GCKD_Genetics_OPN_revised_article_with_changes_highlighted); and 2) a clean version of the manuscript without markings for better readability (GCKD_ Genetics_OPN_Manuscript_clean).

We are looking forward to receiving your response.

Yours sincerely,

Ulla T. Schultheiss

Ulla T. Schultheiss

Institute of Genetic Epidemiology and Department of Medicine IV – Nephrology, Medical Center and Faculty of Medicine – University of Freiburg, Germany,
ulla.schultheiss@uniklinik-freiburg.de; phone: +49 (0)761 270-78210

Reviewer #1: Dear Authors,
In my opinion your corrections increased the value of the manuscript, therefore I believe that the work deserves to be published in the present form.

Reviewer #2: The authors have carefully considered the comments and have reviewed the paper accordingly. The paper is now much more accessible and the findings more clearly displayed.

Reviewer #3: Authors thoroughly addressed previous concerns. The addition of the YFS regional association plots within the main figures is very helpful and nicely supports findings.

One last question is on line 146 "Conditional analysis did not reveal additional independent signals". I assume this means additional signals outside of the three listed regions; however one region (chromosome 4) did exhibit two independent signals (within the same chromosome region). Would suggest better clarifying this in that statement, as identifying two genome-wide significant signals in the same region is very interesting. Biologically this could indicate multiple modes of function regulated by the same locus.

REPLY: The Reviewer is correct in his/her assumption. Conditional analysis just confirmed the existence of the three independent genomic regions that were already visible in the Manhattan plot. Neither outside these three regions nor within these regions were additional signals detected. We revised the respective sentence on page 6 to clarify our statement. It now reads: "*Besides the three identified regions, conditional analysis did not reveal additional independent signals (**S5 Figure**; see page 6, line 146)*."
