# Supplementary material for: Genetics of osteopontin in patients with chronic kidney disease: The German Chronic Kidney Disease study
Source: PLoS Genet. 2022 Apr 6;18(4):e1010139. doi: 10.1371/journal.pgen.1010139 (PMC9015153; doi:10.1371/journal.pgen.1010139)
Supplement: Attachment — Submitted filename: 28JAN22_ReviewerComments.docx [file pgen.1010139.s020.docx]

January 28^th^, 2021

Dear Reviewers,

We thank you for your time and the constructive comments on our manuscript entitled “Genetics of osteopontin in patients with chronic kidney disease: the German Chronic Kidney Disease study” (PGENETICS-D-21-01377R1). We have revised our manuscript according to the points raised and are happy to submit our revised version, which we believe has improved much. A detailed point-to-point response to the comments along with where and how we have altered the manuscript can be found below.

We are submitting two versions of our altered manuscript: 1) a corrected manuscript version with changes marked in track changes (GCKD_Genetics_OPN_revised_article_with_changes_highlighted); and 2) a clean version of the manuscript without markings for better readability (GCKD_ Genetics_OPN_Manuscript_clean). The location of the changes in our point-to-point response refers to the clean version of the altered manuscript and can be found below.

urs sincerely,

Ulla T. Schultheiss

Institute of Genetic Epidemiology and Department of Medicine IV – Nephrology, Medical Center and Faculty of Medicine – University of Freiburg, Germany,

[ulla.schultheiss@uniklinik-freiburg.de](mailto:ulla.schultheiss@uniklinik-freiburg.de); phone: +49 (0)761 270-78210

**Reviewer #1:**

The paper of Cheng and colleagues: “Genetics of osteopontin in patients with chronic kidney disease: the German Chronic Kidney Disease study” presents the results of the genome-wide association study of osteopontin in an European chronic kidney disease (CKD) population.

The manuscript perfectly fits in with quite a number of publications related to the role of OPN and OPN-related genes in chronic disorders, including kidney disease.

The Abstract is well written, accurate, intelligible on its own.

The Author Summary is clear and presents the significance of the obtained results objectively.
The Introduction includes the aim of the study and review of the key literature, however, in my opinion it would also be worth quoting the paper of Kaleta et al. (Cells, 2019) - in line 103.
The Results and methods sections are clear. tables and Figures are accurate and accessible. Results and Methods provide all necessary details to support Authors conclusions.
Discussion is well written and presents the main conclusions, compare the obtained results with the avaliable literature. Moreover, the Authors describe the limitations of their study and plans for future research.

To sum up, the value of the article is great. The paper highlights an interesting topic, and is innovative.

The manuscript is written in a good style and easy to read, there are no grammatical, punctuation or linguistic errors. In the manuscript there are good, clear figures and tables that facilitate understanding of the issues described in the text.

The paper is valuable to publish in PLOS Genetics.

**REPLY**: We thank the Reviewer for the time and effort taken into the review of our manuscript. We appreciate the positive assessment of our work and have incorporated the paper of Kaleta *et al*. (Cells 2019) into the introduction (Reference #14). Please see page 4, line 108 of the clean manuscript version.

=========================================

**Reviewer #2:**

This is a detailed manuscript describing a GWAS in a ckd patient cohort with available osteopontin measurements. Osteopontin is an enigma and this is the first GWAS to investigate it using GWAS. The results however are limited - a locus near the SPP1 gene encoding osteopontin is identified, together with 2 other loci. In a replication cohort, 2 loci were confirmed.

**REPLY:** We thank the Reviewer for the critical assessment of our work.

**Some comments and suggestions**

**Abstract**
"OPN levels are known to be associated with adverse kidney outcomes" - this needs qualification...increased levels?

**REPLY:** We have changed the wording in the abstract, so this fact becomes clearer, as asked by the Reviewer. Please see page 2, lines 49-50 of the clean manuscript version.

**Page 4**

SPP1 gene variants are associated with kidney disease, more detail required here from the 5 refs added

When commenting on the expression patterns of osteopontin (esp in the kidney) important to state which species and how the expression was determined. There are differences between murine rat and human expression.

A potential role for osteopontin in vasculature / atherosclerosis should be mentioned and discussed.

**REPLY:** We have expanded the introduction in order to give more details on *SPP1* gene variants and their association with kidney diseases from the cited references, as asked by the Reviewer. Please see page 4, lines 102-103 and 105-109 of the clean manuscript version. We have also added the respective species to expression patterns of osteopontin, whenever this is mentioned (e.g., page 2, line 68 or page 12, line 296).

Furthermore, we have added a paragraph on osteopontin in cardiovascular disease and atherosclerosis to the discussion. Please see page 15, lines 383-393 of the clean manuscript version.

**Page 5**

Levels of OPN increased n av across the eGFR categories - this is a good example of how the text remains unclear or ambiguous. state it increased from x in CKD stage 1 to y in CKD5

**REPLY:** We have changed the results part to reduce ambiguity of the statement on the increase of OPN levels across CKD stages (Reviewer Table 1), as suggested by the Reviewer. Please see page 5-6, lines 134-137 of the clean manuscript version.

**Reviewer Table 1: Median OPN levels per eGFR and UACR categories**

| **eGFR**  **categories** | **Median OPN**  **(ng/mL)** | **UACR**  **categories** | **Median OPN**  **(ng/mL)** |
| --- | --- | --- | --- |
| G1/2 | 25.4 | A1 | 25.6 |
| G3a | 27.1 | A2 | 30.1 |
| G3b | 31.3 | A3 | 34.2 |
| G4/5 | 38.5 |  |  |

**Page 7**

Some more detail on the relevance of colocalisation studies is required and perhaps a figure to show how the GTEX data integrates with the loci

**REPLY:** To increase understanding we inserted the rationale to conduct colocalization analyses into the Results section (page 7, lines 178-181). Furthermore, we added a new **Figure 3** (referenced on page 8, lines 187-189) illustrating the underlying data that gave rise to the main observed colocalizations using GTEx data. Finally, we additionally included a figure to similarly illustrate the colocalization observed with the UK Biobank phenotype deep venous thrombosis (**S10 Figure**, referenced on page 9, line 220).

The rare variant analysis is interesting. Was this performed in other data sets e.g. UKB

**REPLY:** We performed the rare variant analysis using the GCKD data. Since the implicated gene, *SPP1*, is the OPN-coding gene (Introduction: page 4, 1^st^ paragraph), our finding essentially represents a replication of prior knowledge and thus did not require further replication.

**Page 11**

The ref to osteopontin and nephrolithiasis is from 2007. There is actually very conflicting data surrounding osteopontin and stones - and more update refs and discussion are warranted -eg PMID: 32842990. How does serum osteopontin correlate with urinary osteopontin?

**REPLY:** We have added the suggested reference and enhanced the discussion part on nephrolithiasis and OPN. Please see pages 11-12, lines 289-294 of the clean manuscript version.

I would like to see more figures within the main body of the manuscript, esp those that enable the functional significance of the loci to be determined. Chromatin annotations are not discussed.

**REPLY:** Based on comments from Reviewer 3, we have added additional regional association plots showing the respective results from the replication analysis in YFS cohort (**Figures 1 and 2; panels B**). Furthermore, we have added another figure (**Figure 3**) to the manuscript illustrating the colocalization results we obtained with respect to GTEx data. Both illustrate, obtained evidence from our project. In addition, we provide a new Supporting Table (**S2 Table**, referenced on page 7, line 162; **Methods** described on page 20, lines 488-490), in which we have combined annotations from different sources for the three identified SNPs from GWAS in the GCKD study. Overall, the evidence that these variants play roles in gene regulation is weak and inconclusive and may reflect the inherent limitation of observational studies in which often not the causal variant is/can be observed but some other variants that might be in linkage disequilibrium (LD) with the observed variants.

=========================================

**Reviewer #3:**

Overview:

In “Genetics of osteopontin in patients with chronic kidney disease: the German Chronic Kidney Disease study”, the authors dissect the genetic influences of osteopontin in a large genetic association study. Osteopontin, encoded by SPP1 is implicated with kidney function and the authors hypothesized that a CKD cohort would be ideally suited to identify genetic associations related to the protein. The GWAS of over 3 million genotyped variants (imputed to 7+ million SNPs) yielded three association signals: MEPE-SPP1 (chr 4), KLKB1 (chr 4), and F12-GRK6 (chr 5). The authors tested replication of these signals in the Young Finns Study (a younger, non-CKD cohort) and found evidence of replication for two signals (MEPE-SPP1 and KLKB1). Interestingly, Rare-variant analysis from exome chip SNPs identified a significant variant at a splice-site (rs139555315).

The genetic association methods are well-executed and clearly described within the manuscript.

However, after reviewing, there are several main concerns and several minor suggestions which are listed, below.

**REPLY:** We thank the Reviewer for the critical assessment of our work and helpful comments.

**Main Concerns:**

1) The analysis of OPN in a CKD cohort is a strength of the manuscript, as the authors hypothesize that genetic influences (e.g., SNP associations) may occur within the presence of disease-state (CKD). The authors further support this hypothesis by showing increased OPN levels within greater severity of CKD (e.g, by eGFR category in Figure S2). However, the selected replication cohort (Young Finns Study) is not comparable to the discovery (GCKD) cohort in multiple regards, primarily CKD status (and thus, eGFR). Furthermore, the replication cohort is also different by age and OPN source (plasma versus serum). Since a comparable replication cohort is not available, it seems most appropriate to compare SNP associations across all available OPN-genetics studies (e.g., including those mentioned starting at line 232), perhaps by a summary statistics meta-analysis and/or test of heterogeneity for SNPs in each of the three loci.

**REPLY:** The Reviewer clearly recognizes an important topic. However, we do not only hypothesize that genetic influences may occur in the presence of CKD but – as outlined in our Introduction (page 4-5, lines 109-115) – assume that the setting of CKD may allow assessment of the genetic underpinnings of OPN levels more easily as some of the biological mechanism might be upregulated. Based on our experience in earlier projects (Ref 17-19 in the Manuscript), we found that results obtained from a CKD cohort can reflect both, findings that are transferrable to the general population and thus reflect common physiological features as well as findings that might specifically be due to the underlying disease of CKD.

Ideally, we would have liked to validate our results in an external cohort of similar characteristics and with comparable measurements. Unfortunately, no such study exists, to our knowledge. Among population-based studies, some have measured OPN, but applying various lab techniques. However, different techniques do not provide comparable measurements. In Pietzner *et al.* 2021 (Ref 26 in Manuscript) and in Raffield *et al.* 2020 (Ref 27 in Manuscript), measurements from different platforms were systematically compared showing varying degrees of agreement. In conclusion, a meta-analysis of results for proteins that were measured by different technology is only warranted, if it is known that there is good agreement and overlap. In addition, this would require that all groups had published their results, which is not the case as can be seen e.g. in Sun *et al.* 2018 (Ref 22 in the Manuscript).

The usage of the data from the YFS cohort for validation in our project is not ideal, but seems the best choice among the available studies. Besides being a population-based study with a different sex and age spectrum, it at least has used the same lab technique to measure OPN. Interpretation of replication results was thus done cautiously.

Because of our reasoning above, the findings replicated in YFS cohort may reflect findings in the context of the general population, whereas the unreplicated finding cannot be ignored as it may reflect a CKD-specific finding which we cannot further verify. Overall, our results may require further validation, preferably by functional analyses.

To emphasize this topic more in the paper, we extended the reasoning in the Introduction section (page 4-5, lines 109-115) as well as respective parts in the Discussion section (page 11, lines 265-266 and 268-269).

2) Would suggest evaluating replication of the locus, not just one index SNP. The associations plots show multiple SNPs in high linkage disequilibrium. The causal SNP is not known (and not necessarily the index SNP), thus, would be most advantageous to test all significantly associated SNPs (for each of the three loci) from the discovery cohort in the replication dataset/s.

**REPLY:** Thank you for this good suggestion. We have now generated additional regional association plots for the same regions using YFS results to contrast GKCD findings in a more general context. Each respective figure was added alongside to the figure showing the GCKD results (**Figure 1** and **2, S7 Figure**).

In conclusion, results from both studies align well. For the two replicated loci, the top SNP in the YFS cohort is different but in close proximity and in high linkage disequilibrium with the top SNP observed in GCKD (**Reviewer Table 2**).

**Reviewer Table 2: Top SNPs in GCKD and YFS and their correlation**

| **Study cohort** | **Chr** | **Top SNP** | **Position (GRCh37)** | **p-value** | **R² (D’)*** |
| --- | --- | --- | --- | --- | --- |
| GCKD | 4 | rs10011284 | 4:88833389 | 8.59E-11 | 0.9721  (0.9879) |
| YFS | 4 | rs2126651 | 4:88843810 | 1.47E-05 |  |
| GCKD | 4 | rs4253311 | 4:187174683 | 5.29E20 | 0.9802  (0,996) |
| YFS | 4 | rs143361672 | 4:187157267 | 1.10E-08 |  |

p-value: association p-values from GWAS in respective study cohort

* source: <https://ldlink.nci.nih.gov/?tab=ldpair> (reference population: EUR)

3) Should include GCKD plots that depict the log2(OPN) values by genotype for the three index SNPs (maybe as a supplement).

**REPLY:** Boxplots for log2(OPN) across the whole cohort and per best guess genotypes were generated and added to the Supplement (**S6 Figure**). Its reference in the manuscript can be found on page 6, line 150.

4) While colocalization analysis implicated F11, there is a missed opportunity to highlight that rs4253311 (one of the replicated signals) is an eQTL in multiple tissues for F11 (GTEx V8). So although rs4253311 is intronically located within KLKB1, it might be more appropriate to link it to F11 –which the authors explain F11 is a highly relevant gene for the CKD phenotype.

**REPLY:** We agree with the Reviewer and have added the fact that rs4253311 is an eQTL in multiple tissues for *F11* to the discussion. Please see page 13-14, lines 342-344 of the clean manuscript version.

**Minor Concerns and suggestions:**

1) Text/tables should indicate human reference build (b37 or b38) for SNP positions.

**REPLY:** Thank you for pointing this out. We have now added information on human genome build to the study-specific sections on ‘Genotyping, quality control and imputation’ (GCKD: Methods page 20, line 472; YFS: **S1 Methods**) as well as to all tables and figures in the main manuscript and supplement whenever any genomic position is stated (e.g., **Figure 1**). Almost all human reference builds refer to GRCh37, only results from GTex tissues use GRCh38 (**S3 Table**).

2) Line 140: rs10011284 appears to be upstream (not downstream?) of SPP1, since SPP1 is on the positive strand (UCSC genome browser, b38)

**REPLY:** Thank you for pointing this out. We corrected the manuscript accordingly (Abstract, page 2; Author summary, page 3; Results, page 6, line 151).

3) Would recommend designating loci by their gene name/chromosome versus “1st” and “2nd replicated” locus (lines 248 and 299), gene annotations provide better clarity for the reader.

**REPLY:** We have changed the subheading in the discussion part accordingly. Please see page 11, line 271, page 13, line 327, and page 15, line 371 of the clean manuscript version.

4) For each locus, would recommend including a second association plot that shows SNP signals after adjusting for the index SNP. Given the LD pattern (multiple variants with large p-values and R2 ~0.5 with index SNP) in Figure 1A, am surprised that there was not a secondary signal. A second plot (maybe in supplement) showing the results of the conditional analysis would be helpful.

**REPLY:** In the conditional analysis, we started to repeat analysis for a given chromosome by conditioning on the SNP with the overall lowest p-value in the unconditional analysis. If a genome-wide significant association (p<5E-08) was still present afterwards, the conditional analysis was repeated by adding the next SNP with overall lowest p-value. This procedure was repeated until no further genome-wide significant associations were identified. The results of single steps in this workflow are presented in Reviewer Table 3. In agreement with the Reviewer, we generated step-wise regional association plots for the region with the SNP showing the lowest p-value. The additional plots are added to the Supplement (**S5 Figure**, referenced on page 6, line 147). Furthermore, we revised the respective description in Methods to enhance clarity (Methods page 20, lines 484-487).

**Reviewer Table 3: Step-wise results of conditional analysis**

| **Analysis** | **Condition** | **Chr** | **Top SNP**  **(GRCh37)** | **Position** | **p-value** | **Plot** |
| --- | --- | --- | --- | --- | --- | --- |
| unconditional | - | 4 | rs4253311 | 4: 187174683 | **5.29E-20** | **Figure 1-B**, **New S5 Figure** |
| conditional | rs4253311 | 4 | rs10011284 | 4:88833389 | **9.12E-11** | **New S5 Figure** |
| conditional | rs4253311,  rs10011284 | 4 | rs7655727 | 4: 11424957 | 8.36E-07 | **New S5 Figure** |
| unconditional | - | 5 | rs2731673 | 5:176839898 | **4.5E-25** | **Figure S7**,  **New S5 Figure** |
| conditional | rs2731673 | 5 | rs72759957 | 5: 45286288 | 9.41E-06 | **New S5 Figure** |

p-value: association p-values, bold if <5E-08 (genome-wide significance)

5) In the table of associations, would denote if the SNPs were directly genotyped or imputed.

**REPLY:** We agree that this is a vital information and added a new column to **Table 2** to distinguish genotyped from imputed SNPs. While we used a filter of 0.3 for selection of SNPs analysed in the GWAS, the imputation quality for the 2 imputed SNPs in the GCKD study and for the 3 imputed SNPs in YFS cohort was very good (>0.98). The 3^rd^ SNP in the GCKD study was genotyped.

6) The aggregate testing method was appropriate but surprised authors did not use SKAT-O which is a unified optimal test of both SKAT and the burden test. –By using the SKAT-O method, it would reduce the number of tests (1 test vs 2 per gene). (This is not a requested revision as it would not impact results, here).

**REPLY:** The Reviewer is correct that we could have used SKAT-O, an optimal test that generalizes different settings on genetic architecture into one single test and thereby lessens the burden of multiple testing. Nevertheless, we are interested in the underlying architecture why we preferred to separately conduct the Burden test and the SKAT test. The Burden test also has the advantage of providing effect estimates. This procedure increases the burden on multiple testing and is thus more conservative. Still, we get information on genetic architecture straightaway, at the consequence possibly missing significant associations. As a side note, even if we had only adjusted the significance threshold for only 1 test instead of 2 (0.05/20,000 genes=2.5E-06), no further association would have passed the threshold (min p-value_BURDEN_=3E-05, min p-value_SKAT_=8E-05, excl. results for *SPP1*).

7) Based on the single-variant results (Table 3B), it appears that the splice-variant rs139555314 is likely driving the aggregate test for SPP1. This could be confirmed by running the aggregate test while excluding the splice-variant.

**REPLY:** In our case of *SPP1*, the obtained association statistics of single variants are quite indicative (**Table 3**) that rs139555315 is indeed the variant driving the association for the gene. The additional analysis the Reviewer requested confirms that notion (Reviewer Table 4). This information was added to Results (page 9, lines 236-238).

**Reviewer Table 4: Association results of aggregated variant tests for *SPP1***

| **Analysis** | **No of SNPs aggregated** | **Burden test** | | **SKAT test** | **Total**  **MAC** | **Cumulative**  **MAF of SNPs** |
| --- | --- | --- | --- | --- | --- | --- |
|  |  | **p-value** | **Beta (SE)** | **p-value** |  |  |
| Original analysis (Manuscript **Table 3**) | 7 | 1.36E-03 | -0.31 (0.10) | **2.51E-08** | 59.03 | 6.05E-03 |
| Additional analysis  excluding rs139555314 | 6 | 9.99E-01 | 0.00 (0.11) | 3.52E-01 | 44.01 | 4,51E-03 |

MAC: minor allele count; MAF: minor allele frequency; Statistical significant association p-values are marked in bold (aggregated variant testing: p-value<1.4E-06).
